# Age-Based Differences in Sleep Quality, Pre-Sleep Arousal, and Psychosocial Factors during the Second Wave Lockdown of the COVID-19 Pandemic in Georgia—A Higher Vulnerability of Younger People

**DOI:** 10.3390/ijerph192316221

**Published:** 2022-12-04

**Authors:** Mariam Tsaava, Nikoloz Oniani, Marine Eliozishvili, Irine Sakhelashvili, Nino Tkemaladze, Tamar Aladashvili, Tamar Basishvili, Nato Darchia

**Affiliations:** Tengiz Oniani Laboratory of Sleep-Wakefulness Cycle Study, Ilia State University, Tbilisi 0162, Georgia

**Keywords:** sleep quality, COVID-19 pandemic, pre-sleep arousal, psychosocial, age

## Abstract

The COVID-19 pandemic has deeply disrupted sleep and mental health of people around the world. We aimed to investigate age-based differences in the prevalence of and relationship between sleep quality, pre-sleep arousal, and psychosocial factors during the second wave lockdown of the COVID-19 pandemic in Georgia. Data were collected through an online survey (*n* = 1117). Participants were categorized into four age groups: 18–29, 30–41, 42–53, and 54–70 years. The youngest participants reported the most prevalent disruption of sleep behavior. Overall, 58.3% of respondents were poor sleepers. The Pittsburgh Sleep Quality Index (PSQI) global score was highest in the youngest age group but the difference was not significant. There was a significant difference in the PSQI component scores for subjective sleep quality, sleep latency, and daytime dysfunction, all being worse in young respondents. We also observed a significantly higher prevalence rate of worse sleep quality in the youngest age group, relative to the pre-pandemic period. On the other hand, the oldest respondents showed significantly greater use of sleeping medications. Significantly higher levels of somatic and cognitive pre-sleep arousal, perceived stress, feeling depressed, anxious, and socially isolated were reported by the youngest age group. Study findings indicate a higher vulnerability of younger people to the impact of the COVID-19 pandemic. Assessment of pre-sleep arousal and implementation of specific, age-based interventions may prove beneficial to improve possible consequences of the pandemic on sleep and mental health.

## 1. Introduction

Scientific evidence indicates that the COVID-19 pandemic has altered the lifestyles of billions of people worldwide. Measures undertaken to minimize the spread of the SARS-CoV-2 virus, such as lockdowns, social activity and mobility restrictions, working/studying from home, disrupted daily lives and affected health and well-being of people all around the globe. One of the most frequent complaints during the COVID-19 pandemic period is sleep disturbance [1,2,3]. Several studies investigating the impact of COVID-19 on sleep and mental health of the population across a wide range of countries and across different waves of the lockdown measures have reported a worsening of sleep quality, changes in sleep habits, and high levels of stress, anxiety, and depressive symptomatology [3,4,5,6,7,8]. The persistence of the impact of the COVID-19 lockdown on sleep has also been described [9]. Given that sleep difficulties affect multiple domains of neurobehavioral and physiological functioning, increase the risk for numerous diseases and may have long-term health consequences, managing sleep problems during and after the pandemic waves has been suggested as an important strategy for reducing the pandemic’s global impact on public health [8,10].

Sleep and mental health problems during the COVID-19 crisis and their potential long-term effects on health are important issues for everyone. However, identifying people who are more likely to experience those issues is crucial for developing successful intervention strategies. Several studies have reported that the severity of sleep and psychosocial impairments is higher in the younger population [11,12,13,14,15]. In more detail, sleep problems were more frequently reported in young people aged 18–34 years compared to the elderly in a study of a representative sample of the general population in France [16]. In a longitudinal study carried out in Italy young adults seemed to be the most psychologically affected by the lockdown [17]. The association of younger age with higher stress at follow-up has been found in a 2-month follow-up study of psychological distress in Italy [11]. Marelli et al. [15] demonstrated that the Italian lockdown had a greater impact on sleep and psychoemotional well-being in students than in administrative staff workers. Other studies have also highlighted higher levels of stress, anxiety, and depression in younger populations [18,19].

The majority of studies on age-based differences in the effects of a COVID-19 lockdown are undertaken in groups of only young and elderly individuals, focus on mental health issues, and lack data on sleep behavior. In light of this, the present study aimed to investigate sleep quality, pre-sleep arousal and psychosocial problems, as well as the association between them, among four different age groups, during the second pandemic lockdown in Georgia, when the number of confirmed cases significantly exceeded the case numbers during the first wave [20].

## 2. Materials and Methods

The methods have been published elsewhere [21] and are briefly summarized here.

### 2.1. Study Design and Participants

A cross-sectional online survey was conducted among the general population of Georgia, aged 18 years or older, during the COVID-19 second wave lockdown period. The second and most significant wave of the COVID-19 pandemic started in Georgia at the beginning of the autumn period, 2020. Nationwide two-month restrictions, including a curfew from 21:00 h to 05:00 h, were put in place on 28 November 2020. Beginning on 1 February 2021, lockdown measures started to ease very slowly. The ban on municipal transportation during weekdays was lifted on 8 February 2021 [6]. The data collection took place from 15 January to 8 February 2021. The survey was administered using a Google form hosted by Ilia State University, Georgia. The survey has been disseminated through the University mail list, and on social media (Facebook). In addition, a snowball sampling method was applied, and all participants were asked to disseminate the questionnaire through their social network profiles. The study has been designed in accordance with the Declaration of Helsinki and was authorized by the Ilia State University Research Ethics Committee (#140-35, 14 January 2021). Participants provided online informed consent. From a total sample of 1263 participants surveyed, 1117 valid profiles were analyzed.

### 2.2. Measures

The structured questionnaire consisted of six subsections. The first subsection collected socio-demographic data such as age (categorized into four groups: 18–29 years—group I, *n* = 370, mean age—23.3 years; 30–41 years—group II, *n* = 250, mean age—35.3 years; 42–53 years—group III, *n* = 313, mean age—47.6 years; 54–70 years—group IV, *n* = 184, mean age—57.9 years), sex, marital status (categorized into two groups: married/cohabiting or single/divorced/widowed), education (high school, student, University degree), employment status (employed, unemployed), economic status (bad, average, good), and information on health status (chronic disease, COVID-19 infection). Among those being infected with COVID-19, 19.7% were confirmed by polymerase chain reaction (PCR), and 12.4% were suspected cases based on symptoms and close contact with a diagnosed case, mostly family members. Since there were no significant differences when it comes to the main variables between confirmed and suspected COVID-19 cases, they were categorized into a single COVID-19-infected group. Furthermore, participants reporting having had a coronavirus infection were asked about: (1) whether the infection was ongoing, and (2) the time since the recovery from the disease. None of the participants had an ongoing acute infection or a recovery time equal to or more than 6 months.

The following validated questionnaires were used in the subsequent four subsections.

The Pittsburgh Sleep Quality Index (PSQI) is a widely used questionnaire that consists of 19 items grouped into seven component scores: subjective sleep quality, sleep latency, sleep duration, sleep efficiency, sleep disturbances, use of sleeping medication, and daytime dysfunction. These components are weighted equally on a 0–3 scale (0—no difficulty, 3—severe difficulty). The global PSQI score ranges from 0 (good quality of sleep) to 21 (poor quality of sleep). A global PSQI score of >5 is generally considered to indicate poor sleep quality [22]. The Cronbach’s α of PSQI was 0.80 in the current sample.

The Pre-Sleep Arousal Scale (PSAS) contains 16 items, each rated on a 5-point scale ranging from 1 (not at all) to 5 (extremely). The PSAS measures symptoms of cognitive and somatic arousal experienced at bedtime [23]. The first eight items evaluate somatic pre-sleep arousal (PSAS-somatic), and the last eight items—cognitive pre-sleep arousal (PSAS-cognitive). The clinically relevant cut-off scores for PSAS-somatic and PSAS-cognitive are ≥14 and ≥20, respectively [8,24]. In the present study, the Cronbach’s α for the somatic scale was 0.82, and for the cognitive scale −0.91.

The Perceived Stress Scale-4 (PSS-4) is a brief version of the original PSS-14 instrument designed to assess the respondent’s perception of stress on a 5-point scale from 0 (never) to 4 (very often) [25]. The Cronbach’s α of the PSS-4 in this study was 0.79.

The study instruments (PSQI, PSAS, PSS) showed good validity and reliability in the Georgian population, with details provided elsewhere [26,27,28].

The last subsection included a range of retrospective questions on changes in sleep-wake pattern, sleep quality, and psychosocial variables in the past month (the same reference period as for questionnaires) compared to the pre-pandemic period. Generally, the survey inquired about the changes in bedtime and risetime (delayed, unchanged, advanced); sleep latency, sleep duration, and number of awakenings during the night (increased, unchanged, decreased); sleep quality, access to medical services, and family environment (worse, unchanged, better). Finally, we formulated single ad-hoc questions based on the validated assessment tools (Patient Health Questionnaire-9—PHQ-9; Generalized Anxiety Disorder 7-item scale—GAD-7) to evaluate how depressed (feeling sad, depressed, hopeless, little interest or pleasure in doing things) and anxious (feeling anxious, worrying too much about different things, trouble relaxing) respondents felt during the past month. Each question was scored on a 5-point scale from very low (1) to very high (5). Feeling socially isolated during the past month was also assessed through a single question on a 5-point scale from very low (1) to very high (5).

### 2.3. Statistical Analysis

Demographic, sleep, health, and psychosocial variables were described using counts and percentages for categorical variables and means and standard deviations (SD) for continuous variables. For comparisons, variables were checked for assumptions on the use of parametric and nonparametric tests and then analyzed accordingly. Chi-square, independent-sample t-tests, Kruskal–Wallis H test, Mann–Whitney U test, and analysis of variance (ANOVA) were used. Correlations between variables were tested with Spearman or Pearson correlation, as appropriate.

For hierarchical logistic regression analysis, individuals with PSQI scores >5 were identified as poor sleepers. Block 1 of the regression model included all studied variables as predictors of poor sleep quality, except PSAS-somatic and PSAS-cognitive. Block 2 tested PSAS-somatic and PSAS-cognitive, as additional predictors. In addition, similar regression analyses were conducted separately in each age group. Categorical variables were dummy-coded, and regression models were tested for multicollinearity with a variance inflation factor (VIF). VIF was below 2.5 for all variables. A two-tailed significance level was assumed at 0.05. The statistical analyses were performed using the Statistical Package for Social Sciences (SPSS) version 22.0 (IBM, Chicago, IL, USA).

## 3. Results

### 3.1. Sample Characteristics

Demographic, sleep, health, and psychosocial characteristics of study participants were presented in our initial report from this sample and are briefly summarized here. The mean age of study participants was 38.5 (SD = 13.3; range 18–70) years. Overall, 86.6% of respondents were females, 48.9% were married or cohabiting, 80.8% had a university degree, 71.8% were employed, 61.1% reported an average household economic status, 17.5% had a chronic disease and 32.1% were COVID-19-infected. Demographic, sleep, health, and psychosocial characteristics of study participants are presented in Table 1.

Data are presented as the mean and standard deviation or counts and percentages. PSQI, Pittsburg sleep quality index; PSAS-somatic, Somatic pre-sleep arousal; PSAS-cognitive, Cognitive pre-sleep arousal; PSS-4, Perceived stress scale-4.

### 3.2. Sleep-Wake Patterns

The mean bedtime for the whole sample was 00:50 ± 1:44 h. The difference in bedtime by age groups was significant, with the latest time observed in the younger age group (F_(3,1113)_ = 13.938, *p* < 0.001). Young respondents had significantly longer sleep latency (F_(3,1113)_ = 6.617, *p* < 0.001) and later risetime (F_(3,1113)_ = 56.982, *p* < 0.001). As regards sleep duration, subjective assessment of total sleep time (TST) revealed that younger participants’ sleep duration was significantly higher compared to the other age categories (F_(3,1113)_ = 13.610, *p* < 0.001). Likewise, delayed bedtime, longer sleep latency, and delayed risetime compared to the pre-pandemic period were most frequently reported in the youngest age group (45.9%, 46.2%, and 51.9%, respectively).

Furthermore, a high number of participants (38.9%) reported having worse sleep quality compared to the period before the pandemic, while 3.5% noticed an improvement in their sleep quality. Retrospective assessment of sleep quality showed that a larger proportion of respondents with worse sleep quality were present in the youngest age group (χ^2^(6) = 34.298; *p* < 0.001), and differences between the youngest and the other age groups (45.9%, versus 39.2%, 33.2%, and 33.7%, respectively) were statistically significant at the 0.05 level.

### 3.3. Sleep Quality

**Figure 1:**
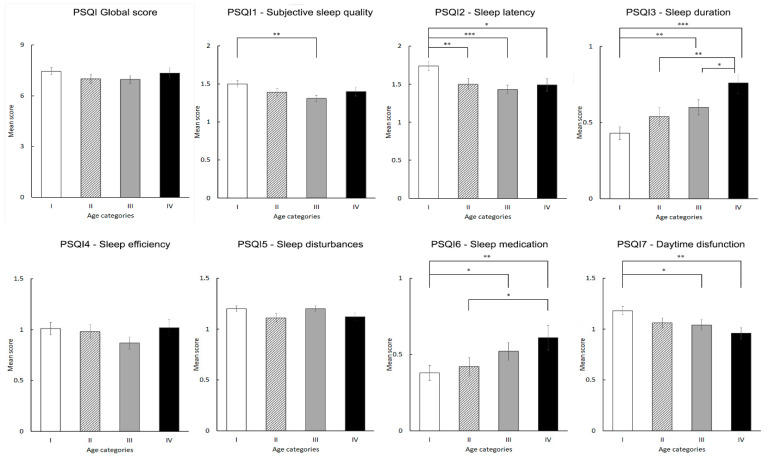
Pittsburg sleep quality index (PSQI) global and component scores according to age groups. * *p* < 0.05; ** *p* < 0.01; *** *p* < 0.001. Age categories: I—18–29 years, II—30–41 years, III—42–53 years, and IV—54–70 years old.

Among the 1117 participants, 58.3% were poor sleepers. The mean PSQI score was 7.19 (SD = 4.17). Although the mean PSQI score was highest in the youngest age group (Figure 1), the difference by age groups was not significant (F_(3,1113)_ = 1.020, *p* = 0.383). The PSQI global score showed the highest correlation with PSAS-cognitive (r = 0.59, *p* < 0.001). Furthermore, PSAS-somatic (r = 0.53, *p* < 0.001), perceived stress (r = 0.40, *p* < 0.001), feeling depressed (r = 0.38, *p* < 0.001), anxious (r = 0.32, *p* < 0.001), and socially isolated (r = 0.21, *p* < 0.01), as well as access to medical care (r = −0.22, *p* < 0.001), having COVID-19 infection (r = 0.16, *p* < 0.001), and family environment (r = −0.15, *p* < 0.001) were significantly correlated with the PSQI global score.

### 3.4. PSQI Component Scores

PSQI1: Subjective sleep quality. Out of the total respondents, 11.8% reported “Very good” sleep quality, 43.3%—“fairly good”, 37.4%—“fairly bad” and 7.4%—“very bad” sleep quality. The highest percentage of “very bad” sleep quality was present in the youngest age group (9.2%, relative to this age category). The difference in PSQI1 component scores between age categories was significant (χ^2^(3) = 10.185; *p* = 0.017), with younger subjects reporting worse subjective sleep quality.

PSQI2: Sleep latency longer than 30 min. Only 18.7 % of respondents did not report difficulties of falling asleep (score 0). The percentage of subjects with sleep latency scores of 2 and 3 was very high—24.4% and 24.9%, respectively. The difference in the PSQI2 component scores by age categories was significant (χ^2^(3) = 16.423; *p* = 0.001). The highest PSQI2 score was most prevalent in subjects aged 18–29 years old (42.4%, relative to this age category).

PSQI3: Sleep duration. The percentage of respondents with an actual sleeping time of more than 7 h was 64.2%; 20.4% scored 1 (6–7 h) and 11.1% scored 2 (5–6 h) on PSQI3, whereas 4.3% reported an actual sleeping time less than 5 h. There was a significant difference in the PSQI3 component scores between age groups (χ^2^(3) = 22.381; *p* = 0.000). The longest sleep duration was reported by the youngest respondents.

PSQI4: Sleep efficiency. Of the total respondents, 45.1% reported sleep efficiency equal to or more than 85%; in 27.8% sleep efficiency was 75–84% and in 12.9% it was 65–74%, whereas 14.2% reported sleep efficiency less than 65%. The difference in the PSQI4 component scores among age groups did not reach significance (χ^2^(3) = 4.641; *p* = 0.200).

PSQI5: Sleep disturbances. Among the studied population, 8.8% had no sleep disturbances; 66.8 % had a sleep disturbance score of 1, 23.3% scored 2, and 1.2% scored 3 on PSQI5. There was no significant difference in the PSQI5 scores between age groups (χ^2^(3) = 5.189; *p* = 0.158).

PSQI6: Use of sleeping medication. Sleeping medication intake was reported by 23.7% of respondents, with the following frequencies of consumption: less than once a week in 9.8%, once or twice a week in 4.9%, and three or more times a week in 9.0%. The age difference in the use of medication was significant (χ^2^(3) = 12.245; *p* = 0.007). The highest proportion of sleeping medication intake was observed in the oldest respondents (11.4%, relative to this age category).

PSQI7: Daytime dysfunction. Among the subjects, 24.4% did not have any trouble with daytime functioning; 49.5% reported difficulties in staying awake and problems in keeping up the enthusiasm to get things done less than once a week, 20.1% reported such difficulties once or twice a week, and 6.0%—three or more times a week. We found a significant difference in the PSQI7 scores between age groups (χ^2^(3) = 9.983; *p* = 0.019) with younger subjects reporting more troubles with daytime functioning.

Differences between PSQI component scores by age groups are presented in Figure 1.

### 3.5. Pre-Sleep Arousal and Psychosocial Variables

**Figure 2:**
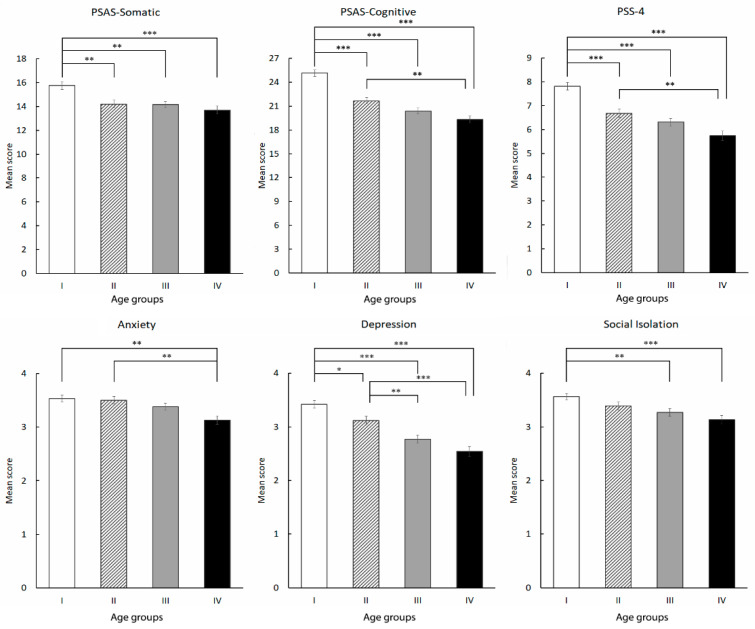
Mean scores of psychosocial variables by age groups. * *p* < 0.05; ** *p* < 0.01; *** *p* < 0.001. PSAS-Somatic, Somatic pre-sleep arousal; PSAS-Cognitive, Cognitive pre-sleep arousal; PSS-4, Perceived stress scale-4. Age categories: I—18–29 years, II—30–41 years, III—42–53 years, and IV—54–70 years old.

As reported in our initial report from this sample, the prevalence of individuals with clinically relevant PSAS-somatic was 49.8%, while with PSAS-cognitive it was 58.0%. The proportion of participants with PSAS-somatic and PSAS-cognitive above the respective cut-off scores differed between the age groups with the highest proportion in the younger age group (57.0% vs. 46.0%, 48.9%, and 41.8% for PSAS-somatic, χ^2^(3) = 13.921, *p* < 0.01; 70.3% vs. 56.4%, 53.4% and 43.5% for PSAS-cognitive, χ^2^(3) = 41.798, *p* < 0.001). Furthermore, PSAS scores decreased significantly across four age categories (Figure 2) from 15.75 to 13.70 (F_(3,1113)_ = 8.627, *p* < 0.001) for PSAS-somatic, and from 25.15 to 19.31 for PSAS-cognitive (F_(3,1113)_ = 40.789, *p* < 0.001). The mean scores for PSAS-somatic (16.64 vs. 11.79, *p* < 0.001) and PSAS-cognitive (25.36 vs. 17.48, *p* < 0.001) were significantly higher in poor sleepers.

The mean PSS-4 score in young respondents was 7.81 ± 0.16 (mean ± se) which was significantly higher compared to the other age categories (F_(3,1113)_ = 27.233, *p* < 0.001; Figure 2).

Overall, a large proportion of respondents reported a high level of feeling anxious (47.0%), depressed (37.3%), and socially isolated (47.2%). Comparing those outcomes between age groups (Figure 2), we found that participants who scored higher on measures of feeling anxious (53.8%), depressed (51.9%), and socially isolated (54.3%) were more prevalent in the youngest age group (*p* < 0.001 for anxiety and depression, *p* < 0.01 for social isolation). Furthermore, mean scores for these variables differed significantly among age groups (Figure 2).

Out of the total sample, 45.6% of respondents reported worsening of the family environment. We found a statistically significant difference between the age groups with proportionally more subjects (51.1%) from the youngest age group reporting worsened family environment (*p* < 0.05). Of the entire sample, 33.9% of respondents reported having more limited access to medical services compared to the pre-pandemic period and this variable did not differ between age groups (*p* = 0.369).

### 3.6. Risk Factors of Poor Sleep Quality

Table 2 presents the results from the hierarchical multiple logistic regression analyses aimed at identifying the potential risk factors for poor sleep quality. Model 1 included socio-demographic and health variables, perceived stress, feeling anxious, depressed, and socially isolated, as well as variables associated with the pandemic-related changes, such as changes in the family environment and access to medical services. Model 2 tested PSAS-somatic and PSAS-cognitive in addition to the variables in Model 1. Both models were statistically significant (*p* < 0.001 for both).

Multiple logistic regression analyses showed that the risk factors of poor sleep quality were age, being a student, COVID-19 infection, perceived stress, anxiety, depression, and social isolation. However, adding PSAS-somatic and PSAS-cognitive to the model identified that being a student, anxiety, depression, and social isolation were no longer significant, while a worsened family environment emerged as a significant predictor (*p* < 0.05), along with somatic and cognitive arousals, both showing a significant relationship with sleep quality (*p* < 0.001 for both). PSAS-somatic and PSAS-cognitive increased the model’s predictive power from 28.6% to 49.0% (Nagelkerke R^2^) and correctly classified 77.7% of the cases. Furthermore, we tested whether the strength of PSAS-somatic and PSAS-cognitive, as predictors of sleep quality, varied between the age groups. The highest, 28.6% increase in predictive power, was found in the youngest age group compared to the other age groups (23.6%, 9.1%, and 20.7% increase in R^2^ in model 2, respectively).

## 4. Discussion

Insufficient sleep is a major societal concern worldwide due to the associated negative health outcomes and safety risks [29,30]. Chronic sleep loss may induce neurobiological changes that are not immediately evident but accumulate over time and may have serious physical and mental health consequences later in life [31,32]. Therefore, the COVID-19 pandemic may have a long-term, wide-ranging impact on people’s sleep, health, and well-being. To the best of our knowledge, this study is the first to assess age-based differences in the prevalence of and the relationship between sleep quality, pre-sleep arousal, and psychosocial factors across a wide age range, among four different age groups, during the pandemic period. Overall, our findings confirm the strong impact of the pandemic on sleep and mental health, especially in younger populations. Our results show: (a) Most substantial changes in sleep pattern relative to the pre-pandemic time in young participants, retrospectively; (b) high prevalence rates of poor sleep quality, clinically significant pre-sleep arousal and psychosocial problems; (b) higher severity of these problems in young participants.

The impact of the COVID-19 pandemic on sleep quality, on the rate of poor sleepers, and sleep profiles across the pandemic waves has been reported in several studies [8,33]. A retrospective assessment of self-reported sleep habits during the second lockdown in a Georgian sample showed that respondents tended to go to bed and wake up later and to have longer sleep latency compared to the pre-pandemic time [26]. Here we report that younger participants were the ones reporting more frequently sleep timing changes and worse subjective sleep quality. It is more likely that disruptions in studying/work schedules due to the pandemic more potently interfere with bedtime habits in younger individuals, which in turn adversely affects sleep quality.

The prevalence rate of poor sleep quality in our study was 58.3%, which is comparable with the results of many previous surveys conducted in different countries during the first wave of the COVID-19 pandemic. In a study conducted in Italy and Belgium, the rate of poor sleep quality was 54.2% and 53.7%, respectively [7]. In another sample of the Italian population, 57.1% of participants reported poor sleep quality [34]. The study of the non-diseased general public during the COVID-19 pandemic in China found that about one-third (36.38%) of participants were poor sleepers [35]. In hospitalized patients diagnosed with COVID-19, 54% suffered with poor sleep quality [36]. In a large-scale study of the Italian population (13989 respondents), over 61% of the participants were poor sleepers [37]. A meta-analysis of the prevalence of sleep problems in populations of 13 countries revealed that sleep problems affected about 40% of people in health care and a comparable rate in general populations. Furthermore, the younger age was associated with a greater magnitude of PSQI-based sleep problems [1].

In the current study, we observed about a 15% increase of individuals with poor sleep quality compared to the rate reported in the non-pandemic period in the Georgian population, indicating a much higher prevalence rate of poor sleepers during the COVID-19 outbreak [27]. Analysis of PSQI component scores in the Georgian population in non-pandemic time showed that sleep problems were more severe in the older population. Even though such comparisons are tentative, we now report that several domains of sleep quality (subjective sleep quality, sleep latency, daytime dysfunction) were more impaired in younger individuals, regardless of the subjectively assessed longer sleep duration. The PSQI assesses multiple facets of sleep. Although it was higher in age group I, PSQI global score did not differ significantly between the age groups. Nevertheless, the comparisons on PSQI component scores highlighted several significant age-related differences. The significantly bad subjective sleep quality, longer sleep latency, and higher daytime dysfunction (all PSQI-based) reported by the youngest participants, as well as the significantly higher prevalence of respondents with a worse sleep quality compared to the pre-pandemic time in the youngest age group, reflect the younger population’s greater level of deterioration in sleep quality domains. Furthermore, the possibility that in young individuals the impact of home confinement on some facets of sleep quality (e.g., sleep disturbances) is hardly detectable in a relatively short period of time, cannot be ruled out. Therefore, the study findings, in line with other studies, are suggestive of a higher vulnerability of younger individuals’ sleep quality to adverse consequences of the pandemic-related lockdowns [1,7,16,19]. On the other hand, the proportion of respondents who used sleep medication in the present study was more than two times higher compared to the previous, non-pandemic results [26], and medication intake was most frequently reported by the older individuals (group IV).

Pre-sleep arousal, with its somatic and cognitive components, is thought to interfere with the ability to fall asleep and maintain sleep [38]. We already described high rates of clinically relevant PSAS-somatic and PSAS-cognitive during the COVID-19 pandemic (49.8% and 58%) which are in line with the results reported by Gorgoni et al. [8]. Further, we showed that PSAS-somatic and PSAS-cognitive were strongly associated with sleep quality (about a 20% increase in R^2^) but psychosocial variables—feeling anxious, depressed, and socially isolated—failed to remain significant in this association in the hierarchical regression analyses. These findings suggest that even though psychosocial variables are strongly associated with sleep quality, they are vulnerability factors for pre-sleep arousal, which most likely mediates these associations. Indeed, evidence supports the idea that somatic pre-sleep arousal represents a marker of anxiety [24]. Furthermore, we have demonstrated for the first time that both components of pre-sleep arousal level are significantly higher, and the predictive power of PSAS-somatic and PSAS-cognitive for sleep quality is stronger in younger individuals, compared to the other age categories. Concerns and uncertainties about the COVID-19, changes in lifestyle, and financial issues may all lead to ruminative thinking/worries, increasing level of pre-sleep arousal and thereby affecting sleep quality, with the strongest effects in younger individuals. At the same time, we observed that perceived stress was a significant predictor for sleep quality as well as for PSAS-somatic and PSAS-cognitive. Bedtime arousal increases in response to stress and mediates stress impact on sleep quality [39,40]. Therefore, we may speculate that pandemic-associated stress is at least partially expressed in the level of arousal during the pre-sleep period, highlighting its role in developing sleep problems during the COVID-19 pandemic. Although we cannot infer causality, the observed age-related differences in perceived stress and pre-sleep arousal level, in light of the apparently bidirectional association between stress, arousal, and sleep [41] require further attention. We agree with Gorgoni et al.’s [8] suggestion that “the evaluation of specific sleep and sleep-related domains like pre-sleep arousal may more easily allow the detection of particular age-related sleep problems during the pandemic than the assessment of global measures of sleep quality”.

The study findings corroborate previous research on sleep and mental health in different waves of the COVID-19 pandemic. The persistent impact of the pandemic on sleep and mental problems across the first and second pandemic waves has been reported in Italy [42]. Conte et al. [33] observed improvement of sleep quality after the first lockdown but further deterioration during the second lockdown. As we already reported, high levels of feeling depressed (37.3%), anxious (47.0%), and socially isolated (47.2%) were found in our study sample [21]. In addition, we report that the most affected group comprised of respondents aged 18–29 years, reporting higher rates of feeling anxious, depressed, socially isolated, and appraising life situations as more unpredictable and stressful. The great impact of the COVID-19 pandemic on university students’ mental health in Georgia during the same pandemic wave has been recently described [43]. Evidence from China suggests that depression and anxiety during the COVID-19 pandemic are associated with higher levels of insomnia in adolescents and young adults [44]. The study of the impact of the COVID-19 pandemic on Australians’ mental health and well-being also observed high levels of negative emotions in young people [45]. Higher vulnerability of younger age groups to stress, depression, and anxiety symptoms during the pandemic has been shown globally (participants from 63 countries) [46]. These data and our study results indicate that the young population represents those with a higher risk of sleep and mental health problems during the COVID-19 pandemic. Therefore, a call for special attention to their health and well-being during and after the several waves of the lockdown and reopening is needed to prevent subsequent emergence of full-blown sleep disorders and/or mental illness. Taken together, study findings emphasize the need for examination of specific age-based differences in multiple domains of sleep and psycho-social variables in order to address sleep and mental health challenges during and after the COVID-19 pandemic and to implement targeted interventions.

Some limitations of the present study should be noted. First, a recruitment bias due to the online survey could have induced underrepresentation of those without Internet access and/or skills. Although in July 2020 the share of households with internet access was 83.8%, and the share of the population aged 15–59 years who uses smartphones was above 90% in Georgia [47], the findings cannot be interpreted as being nationally representative. Next, it is difficult to make causal inferences due to the cross-sectional design of the study. Further, the retrospective assessment of some variables could have led to the recall bias. The evaluation of depression and anxiety with single ad-hoc questions and merging the PCR-confirmed and suspected cases into a single group should also be acknowledged. Finally, the higher proportion of females (86.6%) in this study, as in many online COVID-19 surveys, may have had an impact on prevalence rates of poor sleep quality and clinically relevant pre-sleep arousal.

## 5. Conclusions

Several studies have been conducted to address sleep and psychological problems during the COVID-19 pandemic, but fewer have addressed age group-based differences. In the present study, we found that those aged less than 30 years were at a higher risk of having several sleep quality domains affected and developing a clinically significant pre-sleep arousal level and psychosocial problems. These findings are suggestive of a higher susceptibility of younger people to the changes/disruptions associated with the COVID-19 pandemic. Study findings provide evidence that modulation of pre-sleep arousal (e.g., adaptations of CBT and mindfulness-based therapy elements; relaxation techniques) may counteract the alarming prevalence rates of sleep problems in the pandemic crisis, especially in young populations. Furthermore, policies highlighting the need for age-specific interventions are likely to reduce the impact of the COVID-19 pandemic on sleep and psychosocial variables and prevent long-lasting sleep problems and psychopathologies.

## Figures and Tables

**Table 1:** Demographic, health, and psychosocial variables of the study sample.

	Total Sample *n* = 1117
**Age**	38.50 ± 13.30
**Sex**	
Male	150 (13.4%)
Female	967 (86.6%)
**Marital status**	
Married/cohabiting	546 (48.9%)
Single/divorced/widowed	571 (51.1%)
**Education**	
University degree	902 (80.7%)
High school	59 (5.3%)
Student	156 (14.0%)
**Employment**	
Yes	802 (71.8%)
No	315 (28.2%)
**Economic status**	
Good	213 (19.1%)
Average	683 (61.1%)
Bad	221 (19.8%)
**Chronic disease**	
Yes	196 (17.5%)
No	921 (82.5%)
**Access to medical services**	
Worse	379 (33.9%)
No change	718 (64.3%)
Better	20 (1.8%)
**Family environment**	
Worse	509 (45.6%)
No change	570 (51.0%)
Better	38 (3.4%)
**PSQI global score**	7.19 ± 4.17
**PSAS-somatic**	14.62 ± 5.40
**PSAS-cognitive**	22.07 ± 7.28
**PSS-4**	6.80 ± 2.95
**Anxiety**	3.41 ± 1.17
**Depression**	3.03 ± 1.33
**Social Isolation**	3.37 ± 1.19

**Table 2:** Prediction of poor sleep quality (PSQI >5) based on the logistic regression models.

Predictors	Model 1			Model 2		
	OR	95% CI	*p*	OR	95% CI	*p*
**Age**	1.02	1.01–1.04	0.001	1.04	1.02–1.05	0.000
**Sex**						
Female	Reference					
Male	0.68	0.45–1.03	0.069	0.66	0.42–1.03	0.067
**Marital status**						
Married/cohabiting	Reference					
Single/divorced/widowed	1.14	0.85–1.53	0.392	1.14	0.81–1.59	0.446
**Education**						
University	Reference					
High school	1.54	0.82–2.92	0.183	1.50	0.74–3.05	0.259
Student	1.85	1.09–3.15	0.022	1.47	0.79–2.74	0.227
**Employment**						
Employed	Reference					
Unemployed	0.76	0.54–1.07	0.119	0.83	0.56–1.23	0.352
**Economic status**						
Good	Reference					
Average	1.13	0.79–1.62	0.493	1.07	0.72–1.59	0.739
Bad	0.84	0.53–1.33	0.456	0.87	0.52–1.46	0.588
**Chronic disease**						
No	Reference					
Yes	1.19	0.82–1.73	0.350	1.06	0.70–1.62	0.775
**COVID-19 infection**						
No	Reference					
Yes	1.86	1.38–2.52	0.000	1.64	1.16–2.31	0.005
**Access to medical services**						
No change	Reference					
Worse	1.29	0.96–1.74	0.095	1.24	0.88–1.74	0.213
Better	1.24	0.43–3.54	0.687	1.44	0.44–4.66	0.543
**Family environment**						
No change	Reference					
Worse	1.34	0.99–1.80	0.053	1.45	1.03–2.03	0.032
Better	0.83	0.39–1.76	0.620	0.71	0.29–1.79	0.473
**Anxiety**	1.20	1.03–1.40	0.017	0.96	0.81–1.15	0.685
**Depression**	1.31	1.13–1.51	0.000	1.14	0.96–1.35	0.129
**Social isolation**	1.14	1.00–1.30	0.045	1.12	0.96–1.30	0.148
**PSS-4**	1.25	1.18–1.33	0.000	1.12	1.05–1.20	0.001
**PSAS-somatic**				1.11	1.07–1.16	0.000
**PSAS-cognitive**				1.20	1.16–1.24	0.000
**Nagelkerke R^2^**	0.286			0.490		
**Correct classification (%)**	71.5%			77.7%		

PSQI, Pittsburg sleep quality index; OR, Odds ratio; CI, Confidence interval; PSS-4, Perceived stress scale-4; PSAS-somatic, Somatic pre-sleep arousal; PSAS-cognitive, Cognitive pre-sleep arousal.

## Data Availability

The data that support the findings of this study are available from the corresponding author upon reasonable request.
